# Genome-informed metabolomic re-analysis identifies serum-associated amino acid signatures in sepsis-associated bacterial pathogens

**DOI:** 10.3389/fmed.2026.1734675

**Published:** 2026-06-12

**Authors:** Min Ou, Yulan Luo, Xiaopeng Sun, Shifeng Luo, Mei Feng

**Affiliations:** 1 Department of Critical Care Medicine, West China Hospital, Sichuan University, Chengdu, Sichuan, China; 2 West China School of Nursing, Sichuan University, Chengdu, Sichuan, China; 3 Hemodialysis Center, Chengdu First People's Hospital, Chengdu, Sichuan, China

**Keywords:** antibiotic response, metabolomics, multi-omics integration, serum adaptation, whole-genome sequencing

## Abstract

**Background:**

Antibiotic-resistant bacterial infections remain a major global health threat, particularly in hospital settings. Standard antimicrobial susceptibility testing provides essential clinical information, but it does not fully capture pathogen metabolic adaptation under host-like conditions. Integrating isolate whole-genome sequencing with metabolomics may help prioritize condition-associated metabolic signatures linked to bacterial physiology and antibiotic response.

**Methods:**

We performed a secondary integrative re-analysis of publicly available isolate whole-genome sequencing and matched metabolomics datasets from *Escherichia coli, Klebsiella pneumoniae, Staphylococcus aureus*, and *Streptococcus pyogenes* cultured under RPMI and human serum conditions. Genome assemblies were assessed by quality metrics, fastANI, functional annotation, antimicrobial resistance gene screening, virulence-factor analysis, and KEGG profiling. Metabolomics data were analyzed using PCA, OPLS-DA, KEGG enrichment, and ROC analysis for exploratory feature prioritization. Candidate metabolites were further evaluated in *Klebsiella quasipneumoniae* ATCC 700603 cultured under RPMI or heat-inactivated human serum conditions.

**Results:**

Metabolomic profiles showed condition-associated separation between RPMI and serum samples, with the most consistent serum-associated signature observed in *Klebsiella*. KEGG enrichment prioritized amino acid metabolism, particularly valine, leucine and isoleucine biosynthesis and alanine, aspartate and glutamate metabolism. l-aspartic acid, l-isoleucine, l-leucine, and l-valine showed strong within-dataset discriminatory performance in *Klebsiella*. Experimental validation showed that serum exposure reduced *Klebsiella* growth and viable bacterial burden, increased cell-associated levels of these amino acids, and elevated meropenem and ciprofloxacin MIC-like inhibitory endpoints under serum-conditioned assay conditions.

**Conclusion:**

This integrative re-analysis identified serum-associated amino acid remodeling as a prominent feature of host-like adaptation in Klebsiella. These findings provide candidate metabolic signatures for future mechanistic validation and suggest that serum exposure is associated with growth restriction and reduced antibiotic susceptibility under host-like conditions.

## 1 Introduction

Antibiotic-resistant bacterial infections remain a major global health threat, particularly in hospital settings where multidrug-resistant pathogens contribute substantially to morbidity and mortality (1, 2). Recent global estimates indicate that bacterial antimicrobial resistance directly caused approximately 1.14 million deaths and was associated with 4.71 million deaths in 2021, with the cumulative burden projected to increase substantially by 2050 (3, 4). Among clinically important sepsis-associated pathogens, *Escherichia coli, Klebsiella pneumoniae, Staphylococcus aureus*, and *Streptococcus pyogenes* represent major Gram-negative and Gram-positive bacteria implicated in severe infections (5, 6). Standard antimicrobial susceptibility testing provides essential phenotypic information for clinical decision-making, but it is generally performed under standardized laboratory conditions and does not fully capture the metabolic adaptations that bacteria may undergo in host-like environments (7, 8).

High-throughput omics approaches provide an opportunity to examine pathogen adaptation beyond conventional susceptibility phenotypes. Isolate whole-genome sequencing enables high-resolution characterization of genome content, antimicrobial resistance determinants, virulence-associated features, and functional potential, and has become increasingly important for antimicrobial resistance surveillance and infection control (9, 10). In contrast, metabolomics captures the small-molecule phenotype of bacterial cells and provides a condition-dependent readout of biochemical states shaped by nutrient availability, stress exposure, and host-associated environmental cues (11–13). Whereas genome sequencing defines the genetic repertoire and functional potential of an isolate, metabolomics reflects the biochemical state expressed under a given environment. Integrating genome-derived annotations with metabolite-level phenotypes may therefore help prioritize candidate pathways and metabolic signatures associated with bacterial adaptation under host-like conditions.

Integrated genomic and metabolomic analyses of clinically important bacterial pathogens under host-like conditions remain limited. Here, we performed a secondary integrative re-analysis of publicly available datasets from Mu et al., which profiled clinical isolates of four sepsis-associated bacterial pathogens under RPMI and human serum conditions. We re-analyzed isolate WGS datasets together with matched MetaboLights metabolomics datasets using a unified exploratory framework focused on genome-derived annotations, serum-associated metabolite patterns, cross-species metabolic comparison, and focused experimental validation in *Klebsiella*. Unlike the original study, which primarily characterized conserved and pathogen-specific omics responses, our goal was not to establish clinical diagnostic biomarkers, but to prioritize candidate metabolic signatures of host-like adaptation and experimentally evaluate whether the most consistent serum-associated phenotype was linked to altered growth and antibiotic response in *Klebsiella*.

## 2 Methods

### 2.1 Data acquisition

Publicly available isolate WGS and metabolomic datasets of four clinically relevant sepsis-associated bacterial pathogens, including *Escherichia coli, Klebsiella pneumoniae, Staphylococcus aureus*, and *Streptococcus pyogenes*, were retrieved from public repositories based on the study “Integrative omics identifies conserved and pathogen-specific responses of sepsis-causing bacteria.” For sequencing data, we retrieved publicly available raw paired-end reads deposited in NCBI for *E. coli* (PRJEB29930, *n* = 5), *K. pneumoniae* (PRJEB29928, *n* = 5), *S. aureus* (PRJEB29881, *n* = 5), and *S. pyogenes* (PRJEB29800, *n* = 5). These datasets were generated from cultured bacterial isolates under defined experimental conditions in the original study.

For metabolomic profiling, we obtained datasets from the EMBL-EBI MetaboLights database, which were generated from bacterial cultures grown in both RPMI medium and pooled human serum. Specifically, these included *E. coli* (MTBLS2015, 30 RPMI samples, 30 serum samples), *K. pneumoniae* (MTBLS2322, 12 RPMI samples, 12 serum samples), *S. aureus* (MTBLS1898, 30 RPMI samples, 30 serum samples), and *S. pyogenes* (MTBLS2324, 30 RPMI samples, 30 serum samples). Given the unequal sample sizes across species, downstream discrimination analyses were considered exploratory. All selected datasets contain high-quality, publicly available raw data suitable for exploratory integrative re-analysis. These four species were included because matched publicly available sequencing and metabolomics datasets were available for both RPMI and pooled human serum conditions, and because they represent clinically important sepsis-associated pathogens in the source study.

### 2.2 Assembled genome recovery and quality assessment

Raw sequencing reads for *E. coli, K. pneumoniae, S. aureus*, and *S. pyogenes* were initially assessed using FastQC (v0.11.9; Babraham Bioinformatics, Babraham Institute, Cambridge, UK) to evaluate per-base sequence quality, GC content, and adapter contamination. Reads were then trimmed using Trimmomatic (v0.39; Usadel Lab, RWTH Aachen University, Aachen, Germany) in paired-end mode with the -phred33 setting and the HEADCROP:15 parameter, which removed the first 15 bases from the 5′ end of each read. No host-read depletion was performed because all datasets were generated from bacterial isolate cultures under controlled *in vitro* conditions rather than host-derived clinical specimens. The filtered reads were subsequently assembled *de novo* using Unicycler. Assembly quality was evaluated using QUAST and CheckM. All assemblies exhibited high completeness, low contamination, and overall high assembly quality, meeting the criteria for high-quality genomes and supporting their use in downstream analyses.

### 2.3 Pairwise fastANI analysis and genomic relatedness clustering

To provide comparative genomic context for the analyzed isolate genome assemblies, pairwise average nucleotide identity (ANI) analysis was performed using fastANI. Only isolate genome assemblies that passed the assembly quality assessment described above were included. Because the genomic datasets were derived from cultured bacterial isolates rather than complex microbial communities, each Unicycler-derived assembly FASTA file was treated as one draft isolate genome, including multiple contigs when present; contigs were not artificially concatenated into single-chromosome sequences. All-vs-all pairwise ANI comparisons were conducted separately within each species group using the –ql and –rl options, and ANI matrices were generated using the –matrix option. To visualize within-species genomic relatedness, ANI matrices were displayed as heatmaps with hierarchical clustering based on ANI-derived distances, calculated as distance = 100 – ANI. The clustering was used only to visualize genomic relatedness among isolate assemblies within each analyzed species group and was not interpreted as a formal core-genome phylogeny. Given the limited number of isolates per species, these analyses were used to support within-dataset genomic contextualization rather than species-wide representativeness.

### 2.4 Genome annotation and identification of antimicrobial resistance genes and virulence factors

High-quality assembled genomes were annotated using Bakta to predict coding sequences and assign functional information to genomic features. To characterize antimicrobial resistance determinants, assembled genomes were screened with AMRFinder for the identification of antimicrobial resistance genes (ARGs) based on a curated reference database. In parallel, virulence-associated genes were identified by aligning the annotated genomic sequences against the Virulence Factor Database (VFDB). Together, these analyses generated a comprehensive feature set including predicted coding sequences, ARGs, and virulence factors for each genome. The resulting annotations were used for downstream comparative analyses and association analyses with metabolite profiles.

### 2.5 Functional annotation and KEGG pathway profiling of assembled genomes

High-quality assembled genomes were functionally annotated using Bakta-predicted protein sequences. For each isolate, the protein FASTA file generated by Bakta was submitted to KofamKOALA for KEGG Orthology assignment. KofamKOALA assigns KEGG Orthology identifiers, namely *K* numbers, to protein sequences using HMMER/HMMSEARCH against the KOfam database. High-confidence KO assignments were retained and mapped to KEGG pathways for pathway-level profiling. KEGG pathway profiles were summarized at the isolate-genome level and used to characterize genome-derived functional potential, particularly pathways related to central carbon metabolism and amino acid metabolism.

### 2.6 Metabolomics data analysis and visualization

According to the original study from which the public metabolomics data were obtained, metabolite extraction was performed using an extraction solution containing the internal standards 3 nmol ^13^${\text{C}}_{5}^{15}$N-Valine, 5 nmol ^13^C_6_-Sorbitol, and 2 nmol 1,2-^13^C_2_-Myristic acid. GC-MS and LC-MS datasets were processed and analyzed separately within each analytical platform to avoid direct cross-platform merging of raw abundance values. Within each platform, metabolomics data were normalized by total peak area to reduce differences in overall signal intensity among samples, followed by log_2_ transformation and Pareto scaling prior to downstream multivariate analysis. Because the public dataset did not provide complete batch metadata, injection order information, or pooled quality-control measurements required for explicit batch-effect correction methods such as ComBat or QC-based signal correction, no additional statistical batch correction was applied. Instead, the present re-analysis relied on platform-specific preprocessing and PCA-based inspection of major clustering patterns, while recognizing residual batch effects as a potential limitation. Principal component analysis (PCA) was performed separately for each bacterial strain and analytical platform to evaluate clustering patterns between RPMI-grown bacterial cultures and pooled human serum-treated groups. PCA and subsequent multivariate analyses were performed using SIMCA 14.1 (Sartorius Stedim Data Analytics AB, Umeå, Sweden), and score plots were generated to visualize group separation.

Differential metabolite analysis was carried out using orthogonal partial least squares discriminant analysis (OPLS-DA) implemented in the ropls package. Variables with a variable importance in projection (VIP) score > 1 and false discovery rate (FDR) < 0.05 were considered significant and were reported as candidate condition-associated metabolites. OPLS-DA models were evaluated using *R*^2^ and *Q*^2^ metrics. To assess model stability and reduce the risk of overfitting, permutation testing was performed with 200 random permutations of class labels. Given the exploratory nature of the study and the limited sample size, OPLS-DA results were interpreted as feature-prioritization evidence rather than definitive validation of condition-specific metabolites.

For visualization of differential metabolites, GC-MS- and LC-MS-derived metabolite features were analyzed separately within each analytical platform and were combined only for graphical presentation. Heatmaps were generated using the pheatmap package in R based on the candidate condition-associated metabolites identified from the platform-specific analyses. The annotation scheme was consistent with the PCA plots: blue indicated the RPMI group, red indicated the pooled human serum group, and a left-side annotation bar denoted the analytical platform from which each metabolite feature was derived (red for GC-MS and green for LC-MS). The heatmap color gradient represented scaled relative abundance within each metabolite feature, ranging from blue (low abundance) to white (medium abundance) to red (high abundance).

To identify shared and unique differential metabolites among the four bacterial species, Venn diagrams were generated using the VennDiagram package in R, highlighting the overlaps and specific metabolic signatures across species.

### 2.7 KEGG-based pathway enrichment and selection of pathway-associated metabolites

Candidate differential metabolites identified from the GC-MS and LC-MS datasets were mapped to KEGG compound identifiers after metabolite name and identifier standardization. KEGG pathway mapping was then performed to determine the metabolic pathways associated with these candidate metabolites. The mapped metabolites were further submitted to MetaboAnalyst (14) for metabolite set enrichment analysis and pathway analysis. Enriched KEGG pathways were prioritized according to enrichment significance and biological relevance to central carbon metabolism and amino acid metabolism. Metabolites contributing to the prioritized pathways were retained as pathway-associated key metabolites for downstream abundance visualization and interpretation.

### 2.8 Metabolite-based ROC analysis for within-dataset condition discrimination

ROC analyses were performed using the pROC package in R to evaluate the ability of selected metabolite features to discriminate serum-conditioned bacterial samples from RPMI-grown samples within each species-specific dataset. The binary outcome variable was the experimental culture condition, namely RPMI vs. pooled human serum, and the predictor variables were normalized metabolite abundance values.

Candidate metabolites identified from within-dataset differential metabolite analyses were first evaluated individually by single-metabolite ROC analysis. For each metabolite, AUC, sensitivity, specificity, and ROC curves were calculated to assess its exploratory discriminative ability. When multiple candidate metabolites were evaluated jointly, prediction scores were generated using cross-validated models where sample size permitted, and AUC values were calculated from out-of-fold prediction scores to reduce optimism associated with apparent within-dataset evaluation.

When cross-validation was not feasible because of limited sample size, AUC values were reported as apparent exploratory estimates. These analyses were performed only for exploratory within-dataset feature prioritization and visualization of serum-associated metabolic signatures, and were not intended to establish clinical diagnostic performance or generalizable biomarkers.

### 2.9 Bacterial strain

*Klebsiella quasipneumoniae* ATCC 700603, a member of the *K. pneumoniae* species complex, was used in this study. This strain belongs to the *K. pneumoniae* complex and is reported to produce SHV-18 β-lactamase. It has also been used as a quality-control strain for extended-spectrum β-lactamase production and antimicrobial susceptibility testing.

Bacteria were recovered from frozen glycerol stocks and streaked onto Luria–Bertani agar plates. After overnight incubation at 37 °C under aerobic conditions, a single colony was inoculated into Luria–Bertani broth and cultured overnight at 37 °C with shaking. The overnight culture was centrifuged, washed twice with sterile phosphate-buffered saline, and adjusted to the required inoculum before subsequent experiments.

### 2.10 Serum and culture conditions

Two *in vitro* culture conditions were established: RPMI and human serum. RPMI 1640 medium was used as the control culture condition. The serum condition was established using commercially available heat-inactivated human serum derived from human male AB plasma, USA origin, sterile-filtered, Sigma-Aldrich, catalog no. H3667. According to the manufacturer, this serum is heat-inactivated, sterile-filtered, and derived from human male type AB plasma.

Because the serum used in this study was heat-inactivated, this model was designed to evaluate serum-associated metabolic adaptation rather than complement-mediated bacterial killing. Serum aliquots were stored at −20 °C and thawed on ice before use. Repeated freeze–thaw cycles were avoided. Unless otherwise indicated, bacteria were inoculated into RPMI or serum at the same starting density and incubated at 37 °C under aerobic conditions.

### 2.11 Growth-curve analysis

Bacterial growth under RPMI and serum conditions was assessed by measuring optical density at 600 nm. Briefly, washed overnight cultures were diluted to the same initial bacterial density in RPMI or human serum and transferred into sterile 96-well plates. Medium-only and serum-only wells were included as blanks. Plates were incubated at 37 °C with shaking, and OD600 values were measured at regular intervals using a microplate reader.

For the serum group, OD600 values were corrected by subtracting the corresponding serum-only blank to reduce interference caused by serum turbidity. Growth curves were generated by plotting blank-corrected OD600 values against incubation time.

### 2.12 Sample preparation for targeted cell-associated metabolomic analysis

For targeted cell-associated metabolomic analysis, *Klebsiella* ATCC 700603 was cultured in RPMI or heat-inactivated human serum under identical inoculation and incubation conditions. At the indicated sampling time point, bacterial cultures were rapidly chilled on ice to quench metabolic activity. Bacterial cells were harvested by centrifugation at 4 °C and washed three times with ice-cold phosphate-buffered saline to minimize residual medium- or serum-derived metabolites.

The washed bacterial pellets were extracted using pre-cooled methanol-based extraction solvent. Samples were vortexed, sonicated on ice, and centrifuged at high speed at 4 °C. The supernatants containing cell-associated metabolites were collected and dried under vacuum or nitrogen. Dried metabolite extracts were reconstituted in the initial mobile phase before LC-MS/MS analysis.

Medium-only and serum-only controls were processed in parallel using the same extraction procedure to correct for background signals derived from RPMI or serum. The targeted metabolites included l-aspartic acid, l-isoleucine, l-leucine, and l-valine, which were selected based on KEGG enrichment and ROC analyses. Metabolite abundance was normalized to viable bacterial counts, bacterial biomass, or total protein content, as appropriate.

### 2.13 LC-MS/MS-based targeted metabolomic analysis

Targeted metabolomic analysis was performed using a Waters Xevo TQ-S UPLC-MS/MS system equipped with an ACQUITY BEH HILIC column, 2.1 mm × 100 mm, 1.7 μm. Mobile phase A consisted of acetonitrile/water, 95:5, v/v, containing 10 mM ammonium formate and 0.125% formic acid, pH 3. Mobile phase B consisted of acetonitrile/water, 50:50, v/v, containing the same buffer system.

The gradient program was as follows: 99% mobile phase A from 0 to 1 min, decreased to 65% mobile phase A at 10 min, and returned to 99% mobile phase A from 11 to 15 min for column re-equilibration. The flow rate was 0.4 mL/min, the injection volume was 5 μL, and the column temperature was maintained at 45 °C.

Mass spectrometric detection was performed in both positive and negative electrospray ionization modes. The capillary voltage was set at 2.0 kV, cone voltage at 40 V, source temperature at 150 °C, desolvation temperature at 500 °C, desolvation gas flow at 1,000 L/h, cone gas flow at 150 L/h, and nebulizer pressure at 6.5 bar.

Data were acquired in multiple-reaction monitoring mode. Metabolites were identified by comparison with authentic standards based on retention time and characteristic ion transitions. Peak integration was performed using instrument-compatible analysis software. Relative metabolite abundance was calculated after background subtraction using the corresponding medium-only or serum-only controls.

### 2.14 Viable bacterial enumeration

Viable bacterial burden was determined by colony-forming unit counting. At the indicated time points, bacterial cultures grown in RPMI or human serum were collected and serially diluted tenfold in sterile phosphate-buffered saline. Appropriate dilutions were plated onto agar plates and incubated overnight at 37 °C. Colonies were counted the following day, and bacterial loads were expressed as CFU/mL. When required, CFU values were log_10_-transformed before statistical analysis.

### 2.15 Standard MIC determination

Standard minimum inhibitory concentrations were determined by broth microdilution according to CLSI M07 principles for aerobic bacteria. CLSI M07 provides standardized guidance for broth dilution and agar dilution antimicrobial susceptibility testing, including inoculum preparation, incubation conditions, endpoint interpretation, and quality-control procedures.

Briefly, bacterial suspensions were adjusted to the recommended inoculum and exposed to serial twofold dilutions of the indicated antibiotics in cation-adjusted Mueller–Hinton broth. Plates were incubated at 37 °C for 18–24 h. The MIC was defined as the lowest antibiotic concentration that completely inhibited visible bacterial growth.

### 2.16 Serum-conditioned antibiotic-response assay

To evaluate antibiotic-response phenotypes under serum-associated metabolic conditions, bacteria were first cultured in RPMI or human serum as described above. After serum or RPMI conditioning, bacterial suspensions were exposed to the indicated antibiotics at predefined concentrations. At selected time points after antibiotic exposure, aliquots were collected, serially diluted in sterile phosphate-buffered saline, and plated for CFU enumeration.

Bacterial survival was expressed as log_10_ CFU/mL or as the percentage of surviving bacteria relative to the bacterial burden before antibiotic exposure. This assay was interpreted as a modified antibiotic survival assay rather than a CLSI-standard MIC assay, because RPMI and serum are not standard media for routine MIC determination.

### 2.17 Statistical analysis

All experiments were performed with at least three independent biological replicates unless otherwise stated. Data are presented as mean ± standard deviation or median with interquartile range according to data distribution. Growth curves and antibiotic survival curves were analyzed using two-way analysis of variance or a mixed-effects model, followed by appropriate multiple-comparison correction. Comparisons between two groups were performed using an unpaired two-tailed Student's *t* test for normally distributed data or the Mann-Whitney *U* test for non-normally distributed data. A two-sided *P* < 0.05 was considered statistically significant.

## 3 Results

### 3.1 Assembled genome recovery and quality profiles

**Figure 1:**
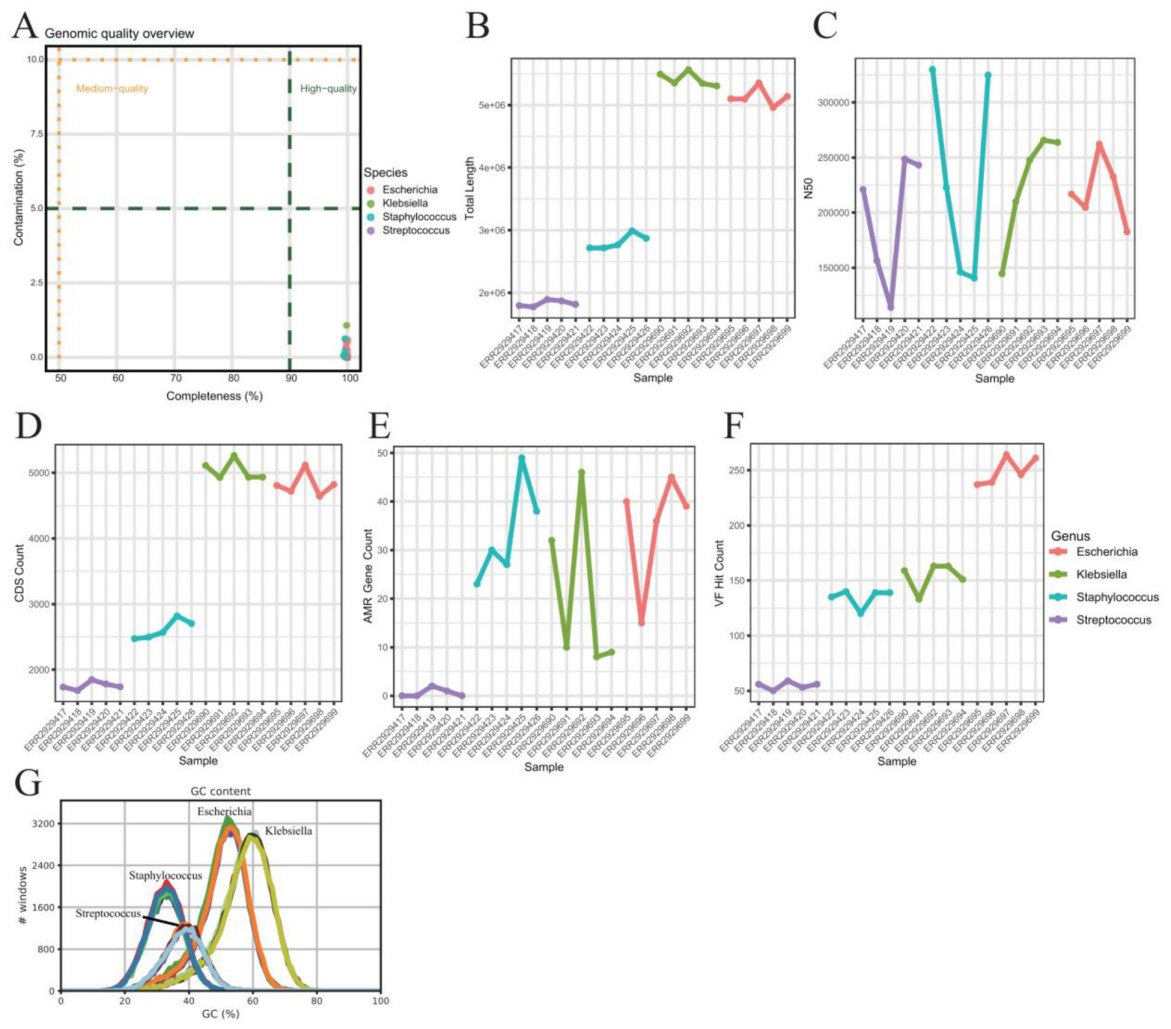
Genome quality metrics and comparative genomic feature distributions across isolate genome assemblies from *E. coli, K. pneumoniae, S. aureus*, and *S. pyogenes*. **(A)** Completeness and contamination of the assembled genomes. Dashed green lines indicate high-quality thresholds (>90% completeness and < 5% contamination), whereas dotted orange lines indicate medium-quality thresholds (>50% completeness and < 10% contamination). **(B)** Total assembly length. **(C)** N50 values. **(D)** Predicted CDS counts. **(E)** AMR gene counts. **(F)** VF hit counts. Each point represents one sample, and colors denote bacterial genera. **(G)** GC content distribution profiles of the assembled genomes across genera. Samples are shown on the *x*-axis in panels **(B–F)**, and colors denote bacterial genera.

Quality assessment showed that most assembled genomes were of high quality, with completeness ranging from 99.32 to 100% and contamination ranging from 0 to 0.08% (Figure 1A). This high assembly quality was further supported by species-consistent genome characteristics across the recovered datasets. Genome size distributions were stable within each species, with *Escherichia* and *Klebsiella* showing the largest total assembly lengths, *Staphylococcus* displaying intermediate genome sizes, and *Streptococcus* exhibiting the smallest assemblies (Figure 1B). A similar trend was observed for predicted coding sequence counts, with higher CDS numbers in *Escherichia* and *Klebsiella* and lower counts in *Streptococcus* (Figure 1D), consistent with the expected differences in genome content among these genera. Assembly contiguity was also generally strong, as reflected by the N50 values across samples, although some inter-sample variation was observed within species (Figure 1C). In addition, GC distribution curves showed clear genus-specific patterns, further supporting the taxonomic consistency and structural reliability of the assembled genomes (Figure 1G). These overall assembly features were further corroborated by circular genome maps, which showed coherent distributions of CDSs, RNA features, GC content, and GC skew across representative genomes from each genus (Supplementary Figure S1).

In addition, downstream annotation revealed clear differences in resistance- and virulence-associated genomic content among genera. AMR gene counts varied markedly across samples, with comparatively low counts in *Streptococcus* and higher but more variable counts in *Escherichia, Klebsiella*, and *Staphylococcus* (Figure 1E). Virulence factor annotations showed a similar species-dependent pattern, with *Escherichia* exhibiting the highest VF hit counts, followed by *Klebsiella* and *Staphylococcus*, whereas *Streptococcus* consistently showed the lowest values (Figure 1F). Together, these results indicate that the recovered genomes were not only of high quality but also retained biologically meaningful interspecies differences in genome size, gene content, GC composition, antimicrobial resistance determinants, and virulence-associated features, supporting their suitability for downstream comparative and integrative analyses.

### 3.2 Pairwise fastANI analysis supports genomic contextualization of isolate assemblies

**Figure 2:**
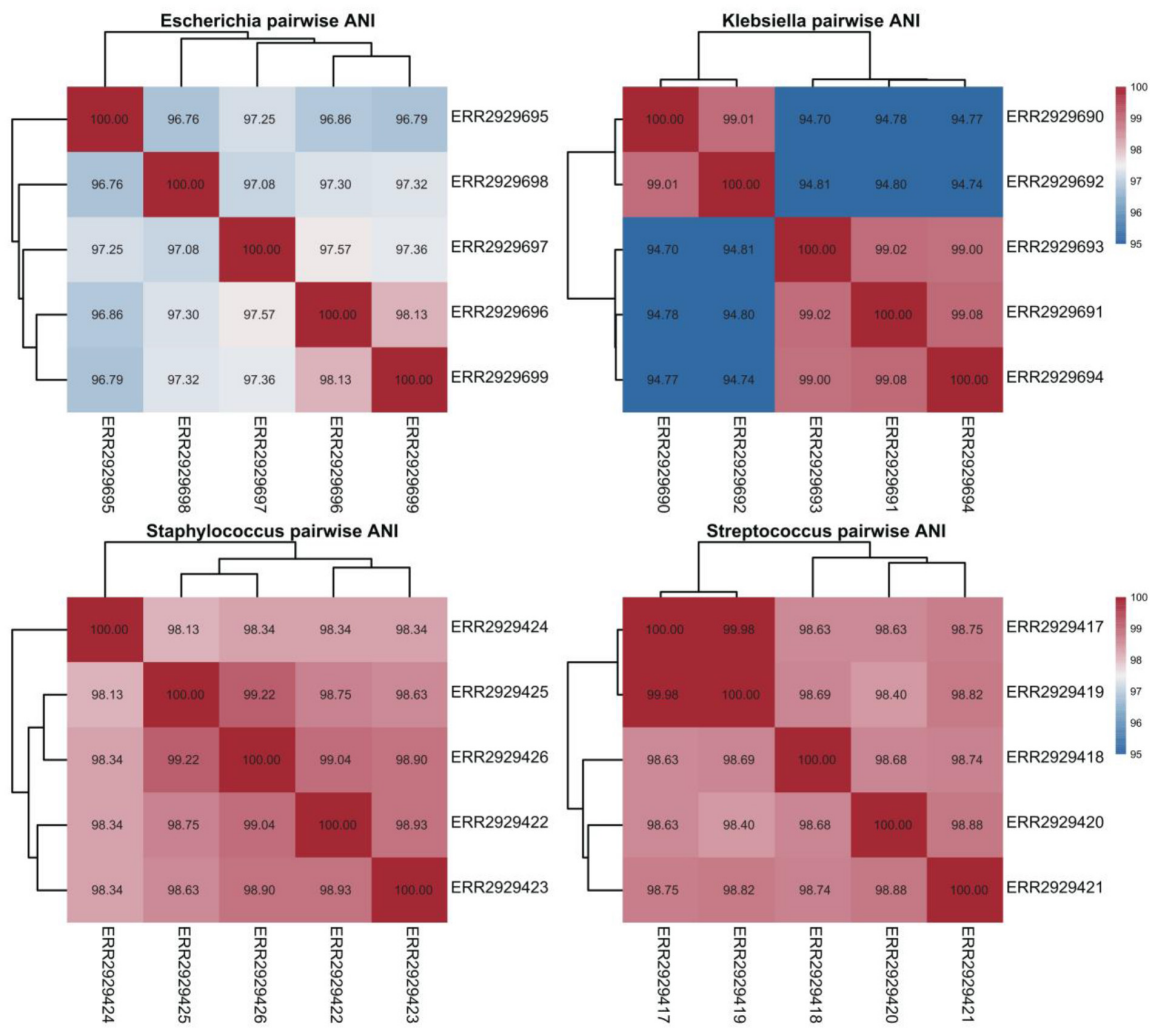
Pairwise fastANI heatmaps of isolate genome assemblies. Heatmaps show all-vs.-all average nucleotide identity (ANI) values (%) calculated using fastANI within each species group. The color scale represents ANI similarity from 95 to 100%, and hierarchical clustering visualizes within-group genomic relatedness. The analysis provides comparative genomic context rather than formal core-genome phylogenetic reconstruction.

Pairwise fastANI analysis was performed to assess genome-level relatedness among the analyzed isolate assemblies within each species group (Figure 2). The *Escherichia* isolates showed consistently high pairwise ANI values, ranging from 96.76 to 98.13%, with the highest similarity observed between ERR2929696 and ERR2929699. These values supported the placement of the analyzed *Escherichia* assemblies within the expected species-level genomic context while indicating moderate within-group genomic variation. In the *Klebsiella* group, pairwise ANI analysis revealed two distinct genomic subclusters: ERR2929690 and ERR2929692 formed one closely related pair (ANI = 99.01%), whereas ERR2929693, ERR2929691, and ERR2929694 formed a second cluster with pairwise ANI values of 99.00–99.08%. By contrast, ANI values between these two subclusters ranged from 94.70 to 94.81%, indicating substantial genomic heterogeneity within the analyzed *Klebsiella* isolate set. The *Staphylococcus* isolates displayed uniformly high within-group ANI values, ranging from 98.13 to 99.22%, with no major divergent isolate detected. Similarly, the *Streptococcus* isolates showed high pairwise ANI values ranging from 98.40 to 99.98%, with ERR2929417 and ERR2929419 showing near-identical genome-level similarity. Overall, the fastANI heatmaps supported close genomic relatedness within the *Escherichia, Staphylococcus*, and *Streptococcus* isolate sets, while highlighting greater heterogeneity in the *Klebsiella* group. Given the limited number of isolates and the borderline inter-cluster ANI values observed in *Klebsiella*, these results were interpreted as comparative genomic context for the analyzed isolate assemblies rather than evidence of species-wide genomic representativeness.

### 3.3 KEGG pathway profiles of protein-coding sequences across four bacterial species

**Figure 3:**
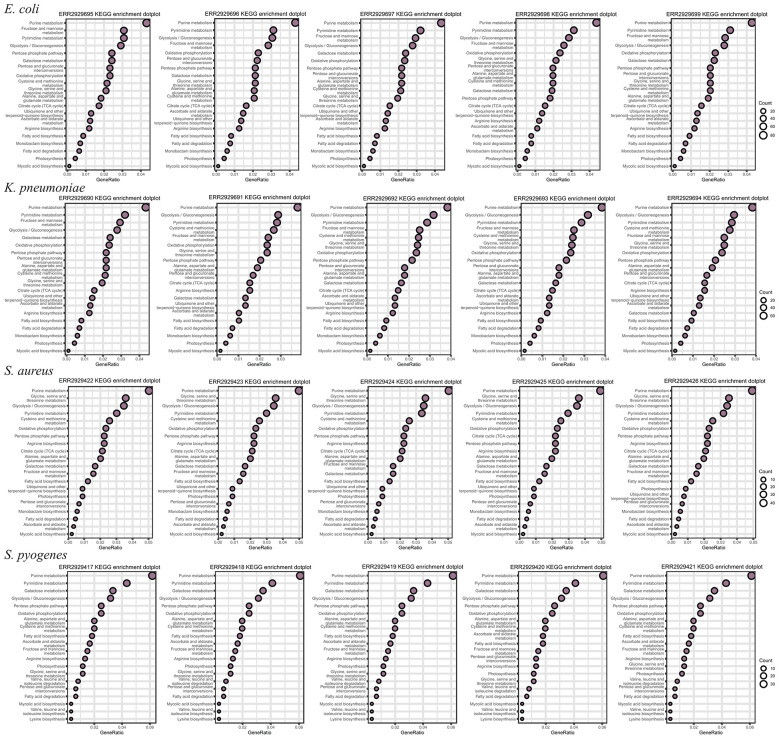
KEGG pathway assignment profiles of annotated protein sequences from four bacterial species. Dot plots show the top KEGG pathways assigned to annotated protein sequences from *E. coli, K. pneumoniae, S. aureus*, and *S. pyogenes* across individual accessions. The *x*-axis represents the GeneRatio, and dot size indicates the number of proteins mapped to each pathway. Across species, the annotated proteins were mainly assigned to pathways related to nucleotide metabolism, central carbon metabolism, oxidative phosphorylation, and amino acid metabolism. These results reflect predicted pathway-level functional composition based on protein sequence annotation rather than pathway activity or differential expression.

KEGG pathway enrichment analysis revealed broadly conserved functional profiles among *E. coli, K. pneumoniae, S. aureus*, and *S. pyogenes* (Figure 3). Across species, the annotated proteins were mainly enriched in purine metabolism, pyrimidine metabolism, glycolysis/gluconeogenesis, pentose phosphate pathway, oxidative phosphorylation, and amino acid metabolism-related pathways, indicating that nucleotide metabolism, central carbon metabolism, and energy production constituted the major functional categories. Species-specific differences were also evident. *E. coli* and *K. pneumoniae* showed highly similar KEGG enrichment patterns, whereas *S. aureus* and *S. pyogenes* exhibited stronger representation of amino acid and carbohydrate metabolism-related pathways. Notably, *S. pyogenes* displayed relatively higher GeneRatio values for several core metabolic pathways, suggesting interspecies variation in the distribution of annotated protein functions.

### 3.4 Differential metabolite profiles show distinct abundance patterns

**Figure 4:**
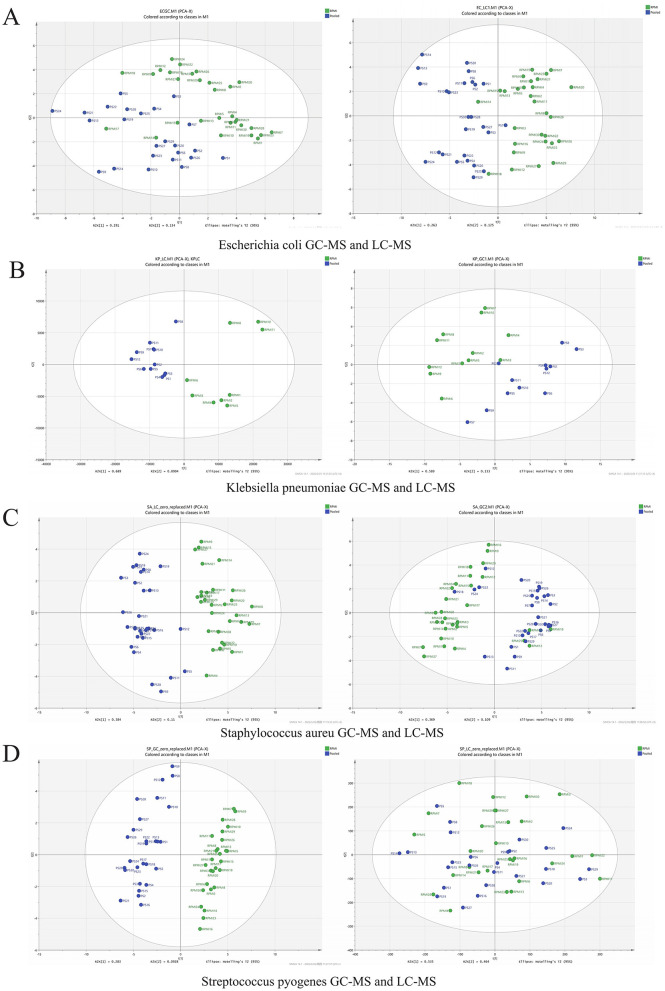
PCA score plots of metabolomic profiles in the RPMI and pooled human serum groups across four bacterial species. **(A)** *Escherichia coli* GC-MS and LC-MS datasets. **(B)** *Klebsiella pneumoniae* LC-MS and GC-MS datasets. **(C)** *Staphylococcus aureus* LC-MS and GC-MS datasets. **(D)** *Streptococcus pyogenes* GC-MS and LC-MS datasets. Green, RPMI group; blue, pooled human serum group. Ellipses indicate Hotelling's *T*^2^ (95%) confidence limits. Percentages indicate the variance explained by each principal component.

PCA analysis revealed species- and platform-dependent differences in metabolite profiles between RPMI-grown and pooled human serum-grown cultures. In *E. coli* (Figure 4A), the two groups showed moderate separation, with more evident separation in the LC-MS dataset than in the GC-MS dataset. The first two principal components explained 29.1% and 15.4% of the variance in the GC-MS data, and 26.3% and 12.5% in the LC-MS data, respectively. In *K. pneumoniae* (Figure 4B), group separation was more pronounced in both analytical platforms, with PC1 and PC2 accounting for 58.9% and 13.3% of the variance in the GC-MS data and 68.9% and 9.04% in the LC-MS data, respectively. *S. aureus* (Figure 4C) also showed condition-associated clustering, with the first two principal components explaining 36.9% and 10.9% of the variance in the GC-MS dataset and 38.4% and 11.0% in the LC-MS dataset. In *S. pyogenes* (Figure 4D), separation was observed in the GC-MS data, where PC1 and PC2 explained 28.3% and 9.28% of the variance, whereas the LC-MS data showed substantial overlap between RPMI and pooled human serum groups despite PC1 and PC2 explaining 53.5% and 46.4% of the variance. Overall, these PCA results indicated that metabolite profiles differed between culture conditions in most species, although the extent of separation varied across bacterial species and analytical platforms. Consistent with the PCA results, platform-specific OPLS-DA score plots showed condition-associated separation between RPMI-grown and pooled human serum-grown cultures across the four bacterial species, with varying degrees of within-group dispersion. These patterns were interpreted as exploratory evidence of condition-associated metabolite profile differences.

Using OPLS-DA modeling together with the combined VIP > 1 and FDR < 0.05 criteria, 112, 169, 116, and 92 candidate condition-associated metabolites were identified for *E. coli, K. pneumoniae, S. aureus*, and *S. pyogenes*, respectively. Model diagnostics were further evaluated using permutation validation (Supplementary Figure S2). Across the GC-MS and LC-MS models, permutation tests showed positive *R*^2^ intercepts and negative *Q*^2^ intercepts, including *E. coli* LC-MS (*R*^2^ = 0.242, *Q*^2^ = −0.485) and GC-MS (*R*^2^ = 0.415, *Q*^2^ = −0.598), *K. pneumoniae* GC-MS (*R*^2^ = 0.179, *Q*^2^ = −0.480) and LC-MS (*R*^2^ = 0.714, *Q*^2^ = −0.913), *S. aureus* GC-MS (*R*^2^ = 0.677, *Q*^2^ = −1.040) and LC-MS (*R*^2^ = 0.132, *Q*^2^ = −0.361), and *S. pyogenes* GC-MS (*R*^2^ = 0.196, *Q*^2^ = −0.388) and LC-MS (*R*^2^ = 0.225, *Q*^2^ = −0.541). These permutation diagnostics provided internal support for model stability, while the resulting metabolite features were interpreted as exploratory condition-associated candidates rather than definitively validated condition-specific metabolites.

### 3.5 KEGG-based pathway prioritization identifies amino acid metabolism-associated candidate metabolites

**Figure 5:**
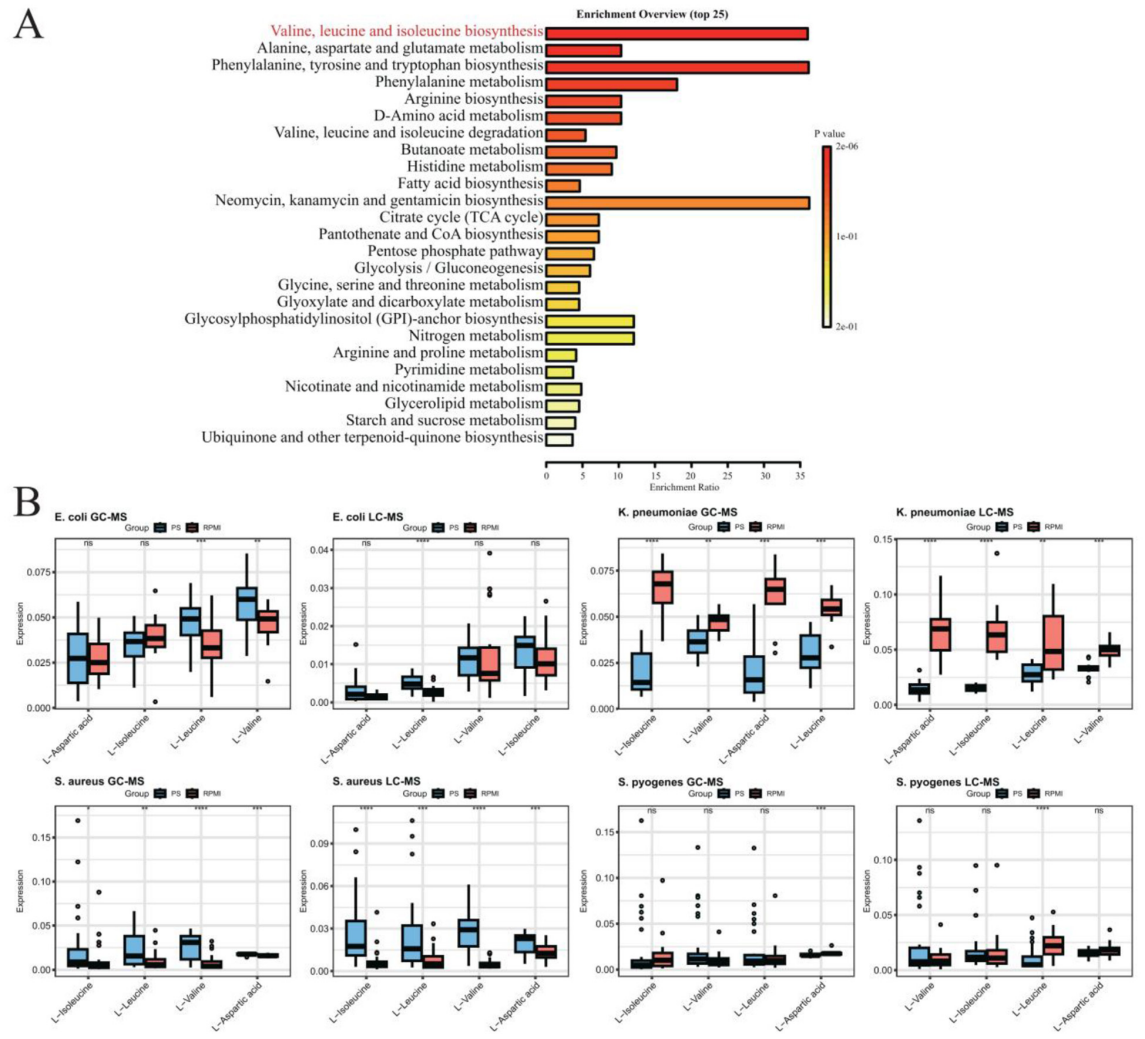
KEGG-based enrichment and abundance distribution of pathway-associated candidate metabolites. **(A)** KEGG enrichment analysis of candidate metabolites. The top 25 enriched pathways are shown according to enrichment ratio, with bar color indicating the corresponding *P* value. **(B)** Relative abundance distributions of KEGG-prioritized metabolites, including l-aspartic acid, l-isoleucine, l-leucine, and l-valine, between PS and RPMI conditions across GC-MS and LC-MS datasets from *E. coli, K. pneumoniae, S. aureus*, and *S. pyogenes*. Boxplots show metabolite abundance differences between groups. Statistical significance is indicated as ns, not significant; *P* < 0.05, *P* < 0.01, *P* < 0.001, and *P* < 0.0001.

KEGG-based enrichment analysis of the candidate metabolite set revealed that amino acid metabolism-related pathways were prominently represented. Among the top-ranked pathways, valine, leucine and isoleucine biosynthesis showed the strongest enrichment signal, together with alanine, aspartate and glutamate metabolism, phenylalanine, tyrosine and tryptophan biosynthesis, phenylalanine metabolism, and arginine biosynthesis (Figure 5A). These results suggest that amino acid metabolism, particularly branched-chain amino acid-related metabolism, represented a major pathway-level feature of the candidate metabolite set. Based on the KEGG enrichment results, four pathway-associated metabolites, including l-aspartic acid, l-isoleucine, l-leucine, and l-valine, were selected for abundance comparison across GC-MS and LC-MS datasets. These metabolites showed clear species- and platform-dependent abundance patterns between pooled human serum (PS) and RPMI conditions (Figure 5B). In *K. pneumoniae*, all four metabolites displayed significant differences in both GC-MS and LC-MS datasets, indicating the most consistent cross-platform response. In *S. aureus*, the selected metabolites were also significantly altered in both platforms, although the magnitude of change varied among metabolites. By contrast, *E. coli* and *S. pyogenes* showed weaker consistency, with only selected metabolites reaching statistical significance in one or both platforms (Figure 5B).

### 3.6 Discriminatory performance of KEGG-prioritized metabolites by ROC analysis

**Figure 6:**
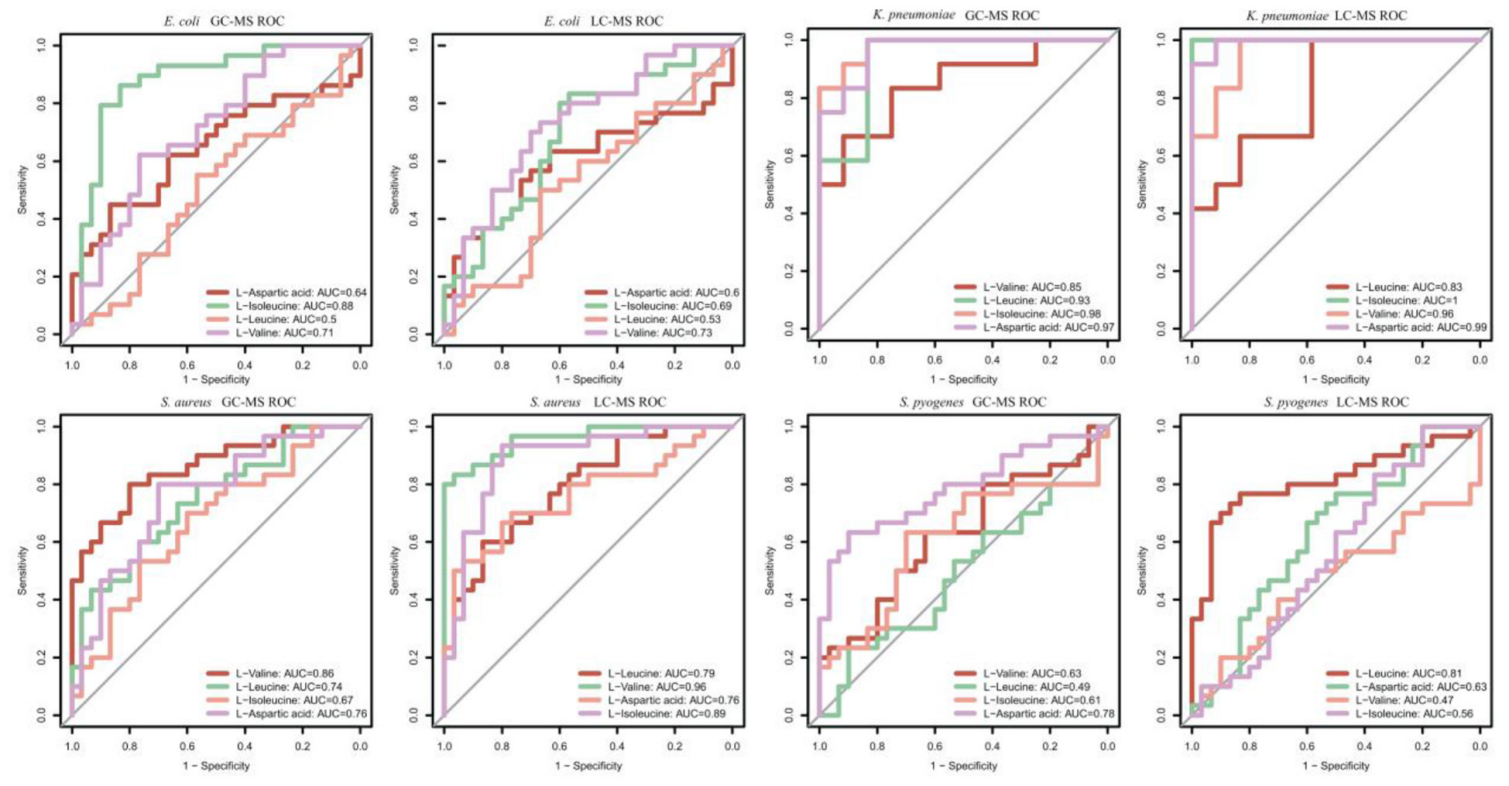
ROC analysis of KEGG-prioritized metabolites across species and analytical platforms. ROC curves showing the discriminatory performance of l-aspartic acid, l-isoleucine, l-leucine, and l-valine between PS and RPMI conditions in GC-MS and LC-MS datasets from *E. coli, K. pneumoniae, S. aureus*, and *S. pyogenes*. AUC values are shown within each plot and were used to evaluate the discriminatory ability of each metabolite.

Among the four bacterial species, *K. pneumoniae* showed the strongest and most consistent discriminatory performance across both GC-MS and LC-MS platforms. In the GC-MS dataset, l-isoleucine, l-aspartic acid, l-leucine, and l-valine showed high AUC values of 0.98, 0.97, 0.93, and 0.85, respectively. Similar performance was observed in the LC-MS dataset, with AUC values of 1.00 for l-isoleucine, 0.99 for l-aspartic acid, 0.96 for l-valine, and 0.83 for l-leucine. In *S. aureus*, the selected metabolites showed moderate to strong discriminatory ability. l-valine displayed the highest AUC in the GC-MS dataset, followed by l-aspartic acid, l-leucine, and l-isoleucine. In the LC-MS dataset, l-valine and l-isoleucine showed stronger discrimination, with AUC values of 0.96 and 0.89, respectively, whereas l-leucine and l-aspartic acid showed moderate performance. By contrast, the discriminatory performance of these metabolites was weaker and more variable in *E. coli* and *S. pyogenes*. In *E. coli*, l-isoleucine showed the highest AUC in the GC-MS dataset, whereas l-valine showed the highest AUC in the LC-MS dataset. In *S. pyogenes*, l-aspartic acid showed the highest AUC in the GC-MS dataset, while l-leucine showed the highest AUC in the LC-MS dataset; however, several metabolites had AUC values close to 0.5, indicating limited discriminatory ability (Figure 6).

### 3.7 Serum reshapes Klebsiella growth and antibiotic susceptibility

**Figure 7:**
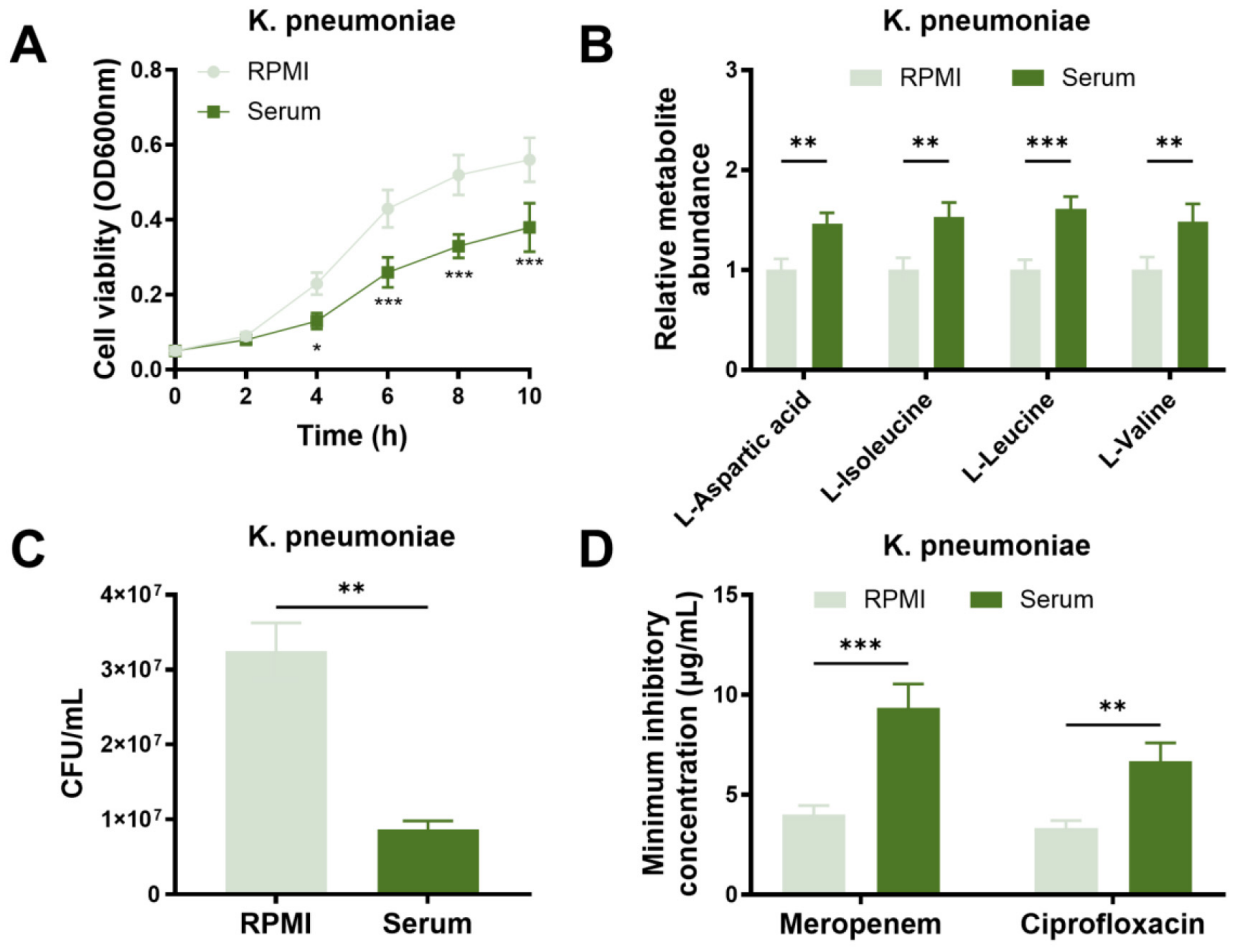
Serum reshapes *Klebsiella* growth and antibiotic response. **(A)** Growth curves of *Klebsiella quasipneumoniae* ATCC 700603 cultured in RPMI or heat-inactivated human serum, assessed by blank-corrected OD600. **(B)** Cell-associated relative abundances of l-aspartic acid, l-isoleucine, l-leucine, and l-valine in washed bacterial pellets from RPMI- or serum-cultured *Klebsiella* ATCC 700603. **(C)** Viable bacterial burden determined by CFU assays. **(D)** Apparent MIC-like inhibitory endpoints of meropenem and ciprofloxacin under RPMI and serum conditions. Data are shown as mean ± SD. Growth curves were analyzed by two-way ANOVA or mixed-effects models; two-group comparisons were analyzed by unpaired two-tailed Student's *t*-test or Mann–Whitney *U* test. ^*^P < 0.05, ^**^P < 0.01, ^***^P < 0.001.

To experimentally assess whether the serum-associated metabolic environment affects Klebsiella physiology, we used *Klebsiella quasipneumoniae* ATCC 700603, a member of the *K. pneumoniae* species complex, as a representative validation strain. Bacteria were cultured under RPMI or heat-inactivated human serum conditions, and bacterial growth, viable burden, cell-associated amino acid abundance, and antibiotic-response phenotypes were evaluated. Growth curve analysis showed that *Klebsiella* ATCC 700603 proliferated progressively in RPMI, whereas bacterial growth was markedly attenuated under serum conditions. Compared with RPMI, serum exposure resulted in significantly lower OD600 values from 4 h onward, and this difference persisted at later time points (Figure 7A). Consistently, CFU enumeration demonstrated a significant reduction in viable bacterial counts under serum conditions, confirming that serum restricted the growth of *Klebsiella* ATCC 700603 rather than merely altering optical density measurements (Figure 7C). Targeted metabolomic analysis of washed bacterial pellets further showed that Klebsiella cells cultured under serum conditions exhibited significantly higher cell-associated levels of l-aspartic acid, l-isoleucine, l-leucine, and l-valine than cells cultured in RPMI (Figure 7B). These metabolites were selected based on the KEGG enrichment and ROC analyses, which prioritized amino acid metabolism, particularly branched-chain amino acid-related pathways, as a major serum-associated metabolic feature. These findings suggest that serum exposure induces a distinct cell-associated amino acid profile in *Klebsiella* cells. We next examined whether serum exposure altered the antibiotic-response phenotype of *Klebsiella* ATCC 700603. In the serum-conditioned antibiotic-response assay, the apparent MIC-like endpoints of meropenem and ciprofloxacin were significantly increased under serum conditions compared with RPMI (Figure 7D). Because RPMI and serum are not standard media for CLSI-defined MIC determination, these results were interpreted as serum-dependent changes in antibiotic-response phenotype rather than standard clinical MIC values. Together, these findings indicate that serum exposure suppresses *Klebsiella* proliferation while inducing cell-associated amino acid remodeling and reducing susceptibility to clinically relevant antibiotics under the tested host-like conditions.

## 4 Discussion

In this study, we performed a secondary integrative re-analysis of publicly available multi-omics datasets from four sepsis-associated bacterial pathogens and combined these analyses with focused experimental validation in *Klebsiella*. The principal finding is that serum exposure was associated with an amino acid-centered metabolic signature, most consistently observed in *Klebsiella*, and that this signature coincided with a phenotype characterized by growth restriction and reduced antibiotic susceptibility under serum-conditioned conditions. These findings suggest that serum does not merely serve as a nutrient source or inhibitory matrix, but provides a host-like physiological context in which bacterial metabolism and antibiotic response are jointly remodeled.

The present work should be interpreted as a hypothesis-generating, validation-oriented extension of the original multi-omics resource generated by Mu et al., which described conserved and pathogen-specific responses of sepsis-causing bacteria under RPMI and pooled human serum conditions (15). Our contribution is not the generation of new global omics datasets, but the re-prioritization of serum-associated metabolic signatures through a genome-informed framework and the experimental testing of a *Klebsiella*-focused phenotype. This distinction is important because pathogen genotype alone does not fully explain bacterial behavior under host-like conditions. While WGS defines functional potential, metabolomics captures the realized biochemical state of bacteria in a specific environment (16).

The genomic analyses provided useful context for interpreting the metabolomic findings. Pairwise ANI analysis showed that *Klebsiella* isolates were more heterogeneous than the other species analyzed, consistent with the known complexity of the *K. pneumoniae* species complex (17). However, despite this genomic heterogeneity, *Klebsiella* displayed the most consistent serum-associated metabolic discrimination. This suggests that serum exposure may impose convergent metabolic pressure on *Klebsiella*, producing a shared condition-associated phenotype across a genomically diverse background. This interpretation supports the value of integrating genome-derived functional potential with metabolite-level phenotypes rather than relying on genome annotation alone.

Amino acid metabolism emerged as the most coherent biological theme. KEGG-based enrichment prioritized valine, leucine and isoleucine biosynthesis, together with alanine, aspartate and glutamate metabolism. Accordingly, l-isoleucine, l-leucine, l-valine, and l-aspartic acid were selected for targeted validation. Branched-chain amino acids are involved not only in protein synthesis but also in bacterial stress adaptation and virulence-associated physiology (18, 19). l-aspartic acid links nitrogen metabolism, nucleotide biosynthesis, and central carbon metabolism (20, 21). The increased cell-associated levels of these metabolites in serum-cultured *Klebsiella* therefore suggest serum-associated amino acid remodeling rather than a simple reflection of extracellular nutrient abundance. Because these measurements represent steady-state abundance, however, they cannot distinguish increased uptake, increased biosynthesis, or reduced utilization.

The most important biological observation is the dissociation between growth and antibiotic response. Serum exposure reduced *Klebsiella* proliferation but increased apparent MIC-like inhibitory endpoints for meropenem and ciprofloxacin. This argues against a simple model in which growth restriction necessarily increases antibiotic susceptibility. Instead, serum-conditioned *Klebsiella* may adopt a physiological phenotype in which bacterial proliferation is constrained while antibiotic response is also altered. This interpretation is consistent with the broader principle that bacterial metabolic state can influence antibiotic efficacy through effects on growth rate, target activity, respiration, membrane potential, and stress-response pathways (22, 23). Prior studies have shown that metabolic perturbations can modify antibiotic killing, particularly in aminoglycoside models (24, 25). Although these findings cannot be directly extrapolated to meropenem or ciprofloxacin, they support the concept that bacterial metabolism can shape antibiotic response.

The antibiotic-response findings should be interpreted as serum-conditioned susceptibility changes rather than standard clinical MICs, because RPMI and serum are not CLSI-defined media for antimicrobial susceptibility testing. They also do not establish classical tolerance or persistence, which require killing-kinetic evidence such as time-kill curves (26), MDK measurements (27), or persister assays (28). Therefore, the most appropriate conclusion is that serum exposure was associated with reduced antibiotic susceptibility under the tested host-like conditions.

Several limitations should be acknowledged. First, the main omics analyses were based on public datasets and were constrained by available sample size, metadata, metabolite annotation, and batch information. Second, genome and metabolome data were integrated at the species and condition level rather than through large-scale matched isolate-specific modeling. Third, the validation experiment used a single *Klebsiella* strain, *K. quasipneumoniae* ATCC 700603, and therefore requires confirmation across diverse clinical isolates. Fourth, the targeted metabolomics data measured steady-state cell-associated metabolite abundance, not metabolic flux. Finally, the antibiotic-response assay measured MIC-like endpoints rather than killing kinetics, limiting conclusions regarding tolerance or persistence.

Future studies should therefore test causality directly. Isotope tracing could determine whether serum-conditioned *Klebsiella* increases amino acid uptake, biosynthesis, or retention. Metabolite supplementation and depletion experiments could assess whether l-isoleucine, l-leucine, l-valine, or l-aspartic acid directly modulate meropenem or ciprofloxacin response. Time-kill assays and persister-frequency measurements are also needed to determine whether serum-conditioned cells display true tolerance-related phenotypes. Validation across a genetically diverse *Klebsiella* isolate panel will be essential to define the generalizability of this serum-associated phenotype.

## 5 Conclusion

Overall, these findings provide an exploratory prioritization of isolate genome-derived features and condition-associated metabolite signals under *in vitro* serum-mimicking conditions. They should be interpreted as hypothesis-generating observations that require validation using biomass-normalized metabolomics, blank medium controls, independent isolate collections, and clinically relevant samples.

## Data Availability

The datasets presented in this study can be found in online repositories. The names of the repository/repositories and accession number(s) can be found at: all sequencing and metabolomics datasets analyzed in this study are publicly available in the NCBI and EMBL-EBI MetaboLights repositories under accession numbers PRJEB29930, PRJEB29928, PRJEB29881, PRJEB29800, MTBLS2015, MTBLS2322, MTBLS1898, and MTBLS2324.
