# Supplementary material for: Genome-informed metabolomic re-analysis identifies serum-associated amino acid signatures in sepsis-associated bacterial pathogens
Source: Front Med (Lausanne). 2026 Jun 12;13:1734675. doi: 10.3389/fmed.2026.1734675 (PMC13307108; doi:10.3389/fmed.2026.1734675)
Supplement: Supplementary file 1 [file Supplementary_file_1.docx]

**Supplementary Figures**


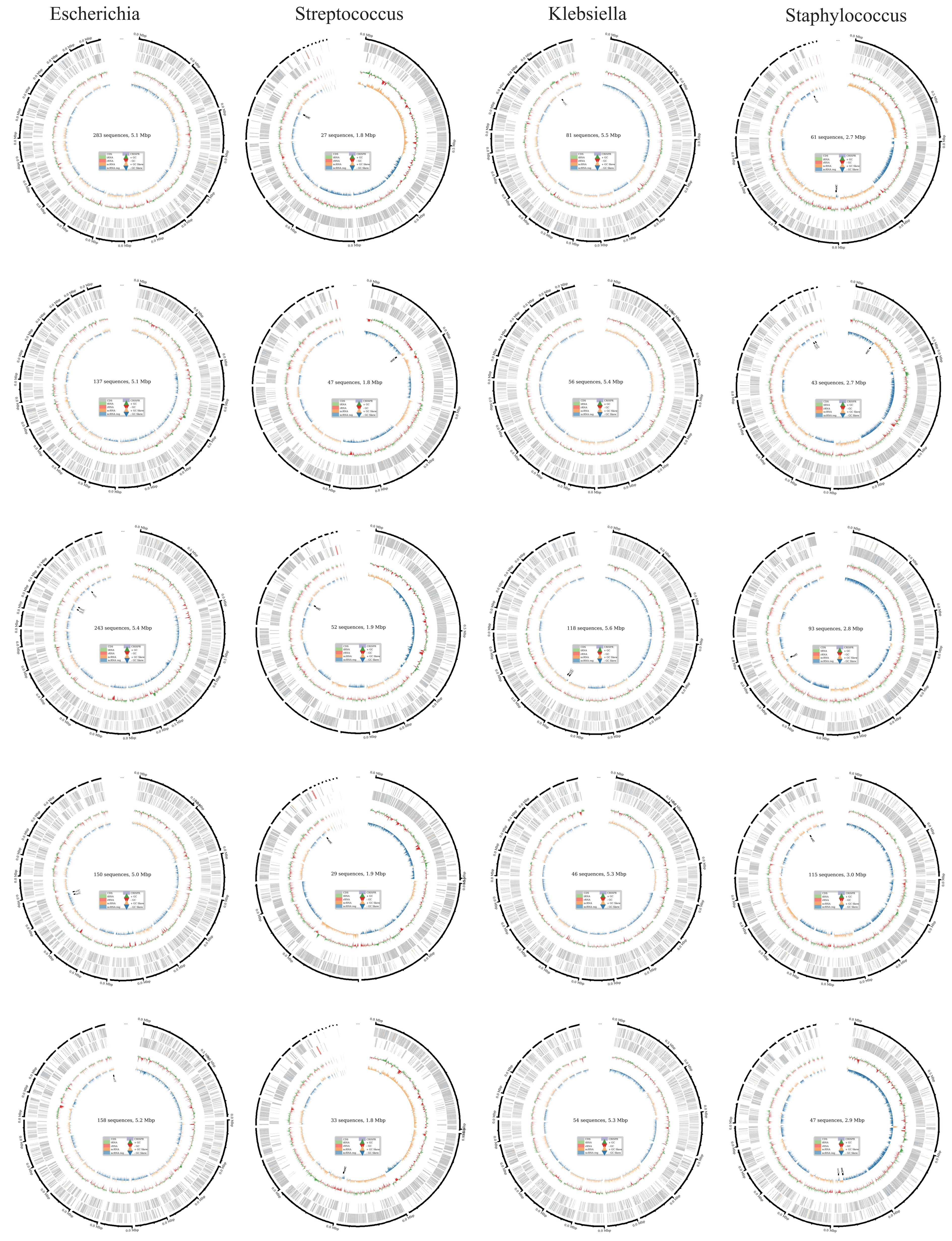


**Figure S1. Comparative circular genomic maps of representative genomes from *Escherichia, Streptococcus, Klebsiella, and Staphylococcus*.** Each panel represents one genome assembly, with five representative genomes shown for each genus. Tracks indicate the distribution of CDSs, tRNAs, rRNAs, ncRNAs, regulatory ncRNAs, CRISPR loci, GC content, and GC skew. The total assembly size and number of sequences are shown in the center of each map.


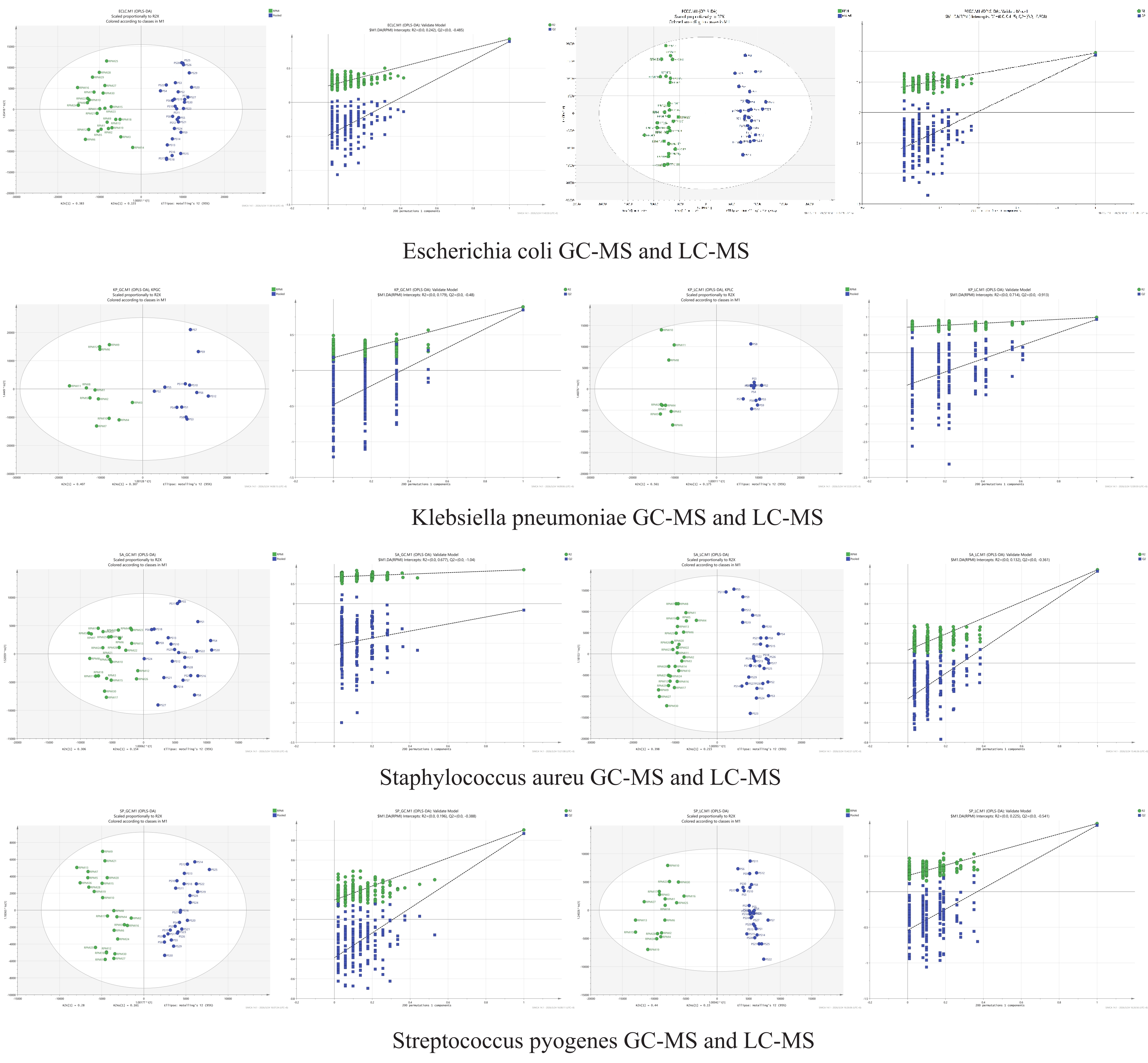


**Figure S2. OPLS-DA score plots and permutation validation plots for the RPMI and pooled human serum groups across four bacterial species.** (A) *Escherichia coli* LC-MS (left two panels) and GC-MS. (B) *Klebsiella pneumoniae* GC-MS (left two panels) and LC-MS. (C) *Staphylococcus aureus* GC-MS (left two panels) and LC-MS. (D) *Streptococcus pyogenes* GC-MS (left two panels) and LC-MS. Green and blue denote the RPMI and pooled human serum groups, respectively, in the score plots. In the permutation plots, green circles represent R² and blue squares represent Q². Ellipses indicate Hotelling’s T² (95%) confidence limits.
